# The Effect of a Flow Field on Chemical Detection Performance of Quadrotor Drone

**DOI:** 10.3390/s20113262

**Published:** 2020-06-08

**Authors:** Sangwon Do, Myeongjae Lee, Jong-Seon Kim

**Affiliations:** CBR Defense Technology Directorate, Agency for Defense Development, Daejeon 34186, Korea; dsw7739@add.re.kr (S.D.); leemj@add.re.kr (M.L.)

**Keywords:** UAV, quadrotor drone, air flow, chemical detection, PIV, carbon nanotube sensor

## Abstract

The determination of a suitable sensor location on quadrotor drones is a very important issue for chemical reconnaissance platforms because the magnitude and direction of air velocity is different for each location. In this study, we investigated a customized chemical reconnaissance system consisting of a quadrotor drone and a chip-sized chemical sensor for detecting dimethyl-methylphosphonate (DMMP; a Sarin simulant) and investigated the chemical detection properties with respect to the sensor position through indoor experiments and particle image velocimetry (PIV) analysis of the system. The PIV results revealed an area free of vortex–vortex interaction between the drone rotors, where there was distinctly stable and uniform chemical detection of DMMP. The proposed chemical reconnaissance system was found to be realistic for practical application.

## 1. Introduction

Quadrotor drones have become popular, finding application in various areas such as security [1], surveillance [2,3,4], rescue [5], and terrestrial exploration [6,7] owing to their mobility, ability to access confined spaces (e.g., caves, buildings, and bunkers), and availability. They play an important role in state-of-the-art transport and technological advances have enabled the integration of various types of sensors for the substitution of humans in optical [8,9,10,11,12] and olfactory [13,14,15,16] sensing. However, the application of quadrotor drones to chemical sensing has encountered relatively greater limitations owing to issues such as air flow fluctuation and chemical adsorption [17,18]. This has necessitated further aerodynamic investigation and realistic demonstration toward improving the feasibility of quadrotor drones for chemical reconnaissance.

**Figure 1:**
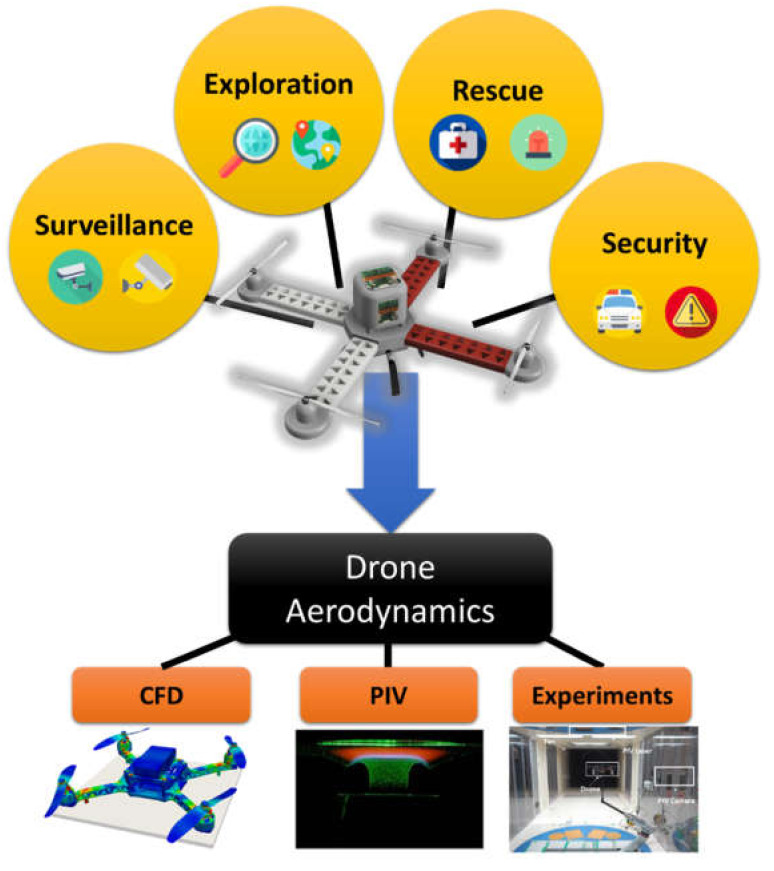
Drone development requires aerodynamic investigation by computational fluid dynamics (CFD), particle image velocimetry (PIV), and realistic experimentation.

As illustrated in Figure 1, there are several methods for conducting aerodynamic investigations of quadrotor drones, such as computational fluid dynamics (CFD), particle image velocimetry (PIV), and realistic experimentation. CFD involves calculation of the flow field around the drone using turbulence models and drone grid-based 3D modeling. PIV involves direct observation of the flow field around the drone and is the most commonly used method for air frame flow measurement. While CFD enables aerodynamic prediction through computer simulations and PIV facilitates direct measurement of the flow field, realistic experimentation enables demonstration of the aerodynamic phenomena observed by CFD or PIV. Realistic experimentation represents a large hood test or outdoor test with rotor operation.

Various studies have been conducted to analyze the air flow field of quadrotor drones by CFD [19,20,21,22], PIV [23,24,25], and realistic experimentation [26,27], and the occurrence of a strong down flow has been identified as a major aerodynamic issue in the practical application of chemical detection using quadrotor drones [27,28].

All the flight controls of a quadrotor drone are based on variation of the speeds of the four rotors. Hence, a strong down flow occurs during the flight of a quadrotor drone, resulting in vortex and wake phenomena [29], which could interfere with the detection capability of an integrated chemical sensor. Moreover, periodic flow, introduced by the rotors, can impact the performance of chemi-electronic sensors which have a detection principle based on the chemical adsorption phenomena between target gas molecule and sensing channel [30].

Blade-vortex interaction (BVI) noise can also be a concern, which is introduced by unsteady pressure fluctuations on a blade due to interactions with previously generated tip vortices during descent or maneuvering flight [31]. CFD studies of BVI have also demonstrated that the phenomenon significantly affects the ambient pressure, velocity field, normal force on the surrounding, and acoustic noise [32,33,34,35,36,37]. Experimental observations have confirmed these effects under the actual flight conditions of a single copter-type propeller [38,39]. The results of CFD investigations have likewise been confirmed by experimental method measurements, indicating that BVI phenomena occur around single copter-type propellers, double coaxial copter-type propellers, multi-rotors, and quad-tilt rotors [38,39,40,41,42]. Kok et al. suggested that the turbulence that occurs around the strong down flow under a rotor interferes with a chemical plume tracing (CPT) algorithm, obstructing its function in the detection of chemical agents [22,43]. For these reasons, the aerodynamics around the drone must be considered for quadrotor drone chemical detection.

With increasing interest in UAVs globally, phenomena directly related to their operation have also attracted broad attention over the last decade [44,45]. According to the Seneviratne’s group survey research, it is increasing to interest in drone aerodynamics and the integration of UAVs and chemical agents or sensors [45]. Various analytical studies were conducted during the last decade toward developing a highly efficient chemical detection platform based on a quadrotor drone. Hansen et al. proposed a low-cost and flexible UAV system for the deployment of sensors [46]. Javey et al. investigated the performance of a multiplexed gas sensor attached to a drone [47]. Jordan at al. considered the need of military-related agencies for a quadrotor drone-based decision support tool for the Stryker NBC RV [48]. Neumann et al. developed a chemical hazardous source localization method that utilizes a quadrotor-drone [49,50].

Although the findings these previous studies contribute to the application of drones to chemical detection such as in terrestrial military exploration, very few of the studies involved the combined use of empirical and simulation methods. In our study, the flow around the quadrotor drone during hover flight was visualized by PIV to obtain the velocity field in the segmented flight environment. By considering the results, four different points on the drone were identified as candidates for the attachment of the chemical sensors. To assess the feasibility of the drone–sensor system for chemical reconnaissance, DMMP exposure experiments were performed in a walk-in hood system.

## 2. Materials and Methods

### 2.1. Drone Platform

**Figure 2:**
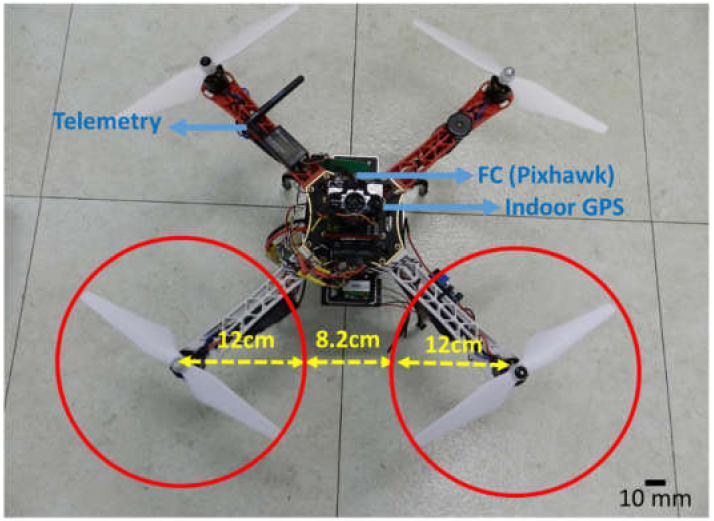
Top view of the customized quadrotor drone consisting of a telemetry system, precise indoor GPS system, and Pixhawk flight controller.

The utilized UAV platform was a Pixhawk quad X quadrotor (http://pixhawk.org) with a fully equipped width of 69 cm, height of 25 cm, and weight of 1.8 kg. It was fitted with an open-source PX4 (http://px4.io) flight control system for autonomous flight along pre-prepared flight paths and management of the entire flight control system. QgroundControl (v. 3.5.2.) was used for the pre-flight management, and rotor control to monitor the flight. A custom program was used to control the rotational speed of each rotor by the pulse width modulation method, with modification of some of the PX4 codes. The communication frequency between the drone and GCS was 915 MHz, while the RC transmitter/receiver frequency was 2.4 GHz. The drone platform was fitted with an indoor GPS system for precise position recognition with a communication frequency of 433 MHz. The quadrotor had a diagonal wingspan of 450 mm (i.e., the distance between the extremities of opposite rotors) with a square body of side 125 mm. The aerodynamics variables, namely the rotor axis-to-axis distance, rotor-to-rotor distance, and rotor blade radius were 32.2, 8.2, and 12 cm, respectively (see Figure 2). Detail specifications are summarized in Table 1.

### 2.2. CNT Sensor

**Figure 3:**
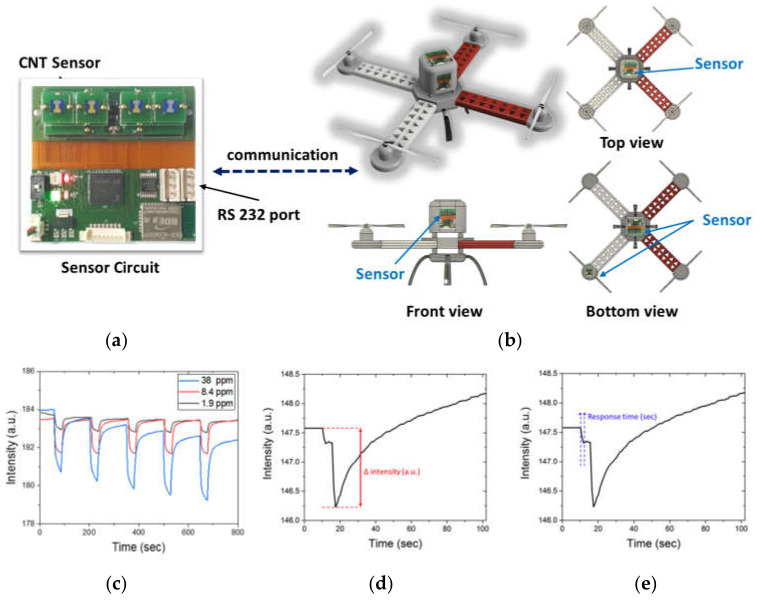
(**a**) Carbon nanotube (CNT) array sensor circuit board showing telemetry communication circuit with four sensor adapters, and (**b**) schematic of a quadrotor drone showing different attachment points of CNT sensors (top, bottom, middle, and on-rotor). (**c**) Capacitance change graphs with different dimethyl-methylphosphonate (DMMP) concentration exposure (1.9, 8.4 and 38 ppm) from laboratory scale test. (**d**,**e**) Change of intensity and response time from our experiments for comparison of each locations’ sensor performance, respectively.

Figure 3a shows the chemi-capacitance sensor circuit for DMMP detection consisting of four CNT sensor sockets, an RS232 communication module, and an LED display for detection feedback. Figure 3b shows a 3D schematic of the quadrotor drone with the attached sensor used in this study. The sensor is detachable and communicates with the drone flight controller in real time, via a cable port. Figure 3b also shows the different possible attachment points of the sensor as determined from the PIV results.

In our experiment, a commercially sourced chip-sized chemi-capacitance sensor consisting of carbon nanotube (CNT) and organic functional groups with a high affinity for the dimethyl-methylphosphonate (DMMP) molecule was installed on the quadrotor drone [51]. The organic functionalized CNT sensors were purchased from SensorTech. Inc. (Republic of Korea) and tested basic DMMP detection performance in small scale laboratory of the company. Figure 3c shows the result of basic DMMP detection performance of sensors with various concentrations (1.9, 8.4 and 38 ppm). As described in Figure 3c, CNT sensor showed fast response time and repeatable performance with DMMP exposure. Because a DMMP molecule contains an organophosphonate group, which has a high molecular polarity and an affinity for the organic function group in a CNT bundle. Hence, the capacitance of a CNT bundle substantially changes on exposure to DMMP gas. This sensor system was integrated with a customized transmitter circuit board for transmission of the detection signals.

We measured capacitance change and response time of our experiments as described in Figure 3d,e, respectively. Figure 3d shows the sensor intensity of CNT sensor which used in the sensor performance tests. In this case, the sensor is attached to Middle position of drone and the *X*-axis of this graph means operating time. The capacitance change (Δ intensity) means the difference between initial sensor intensity and the minimum sensor intensity after sensing DMMP. Response time was measured by calculating the initial detection time of DMMP upon entry into the gas zone to first extreme minimum capacitance of DMMP signal.

### 2.3. Air Flow Visualization by PIV

PIV enables quantitative tracking of fluid motion by illuminated digital imaging with tracer particles of the same specific weight as the fluid injected into the flow field [52]. Pairs of time-spaced images are captured at regular intervals with synchronization of the light source with the camera. The vectors can then be obtained based on the difference between the times and the distances covered by the tracer particles in the two images. The PIV equipment includes a laser light source, CCD camera, and synchronizer, as well as tracking particles and a particle generator.

In this study, the PIV investigation was conducted in a mid-sized subsonic wind tunnel at the Agency for Defense Development, Korea. The process enabled determination of the velocity field distribution around the drone. The laser source was a Nano L200-15 PIV (Litron Laser). The laser was a dual pulse laser that can produce up to 200 mJ of light with a wavelength of 532 nm. The repetition rate of the laser was up to 15 Hz. Di-ethyl-hexyl-sebacate (DEHS) tracer particles were employed for the experiment. The seeding system consisted of an air compressor or high-pressure container that could atomize the liquid DEHS.

**Figure 4:**
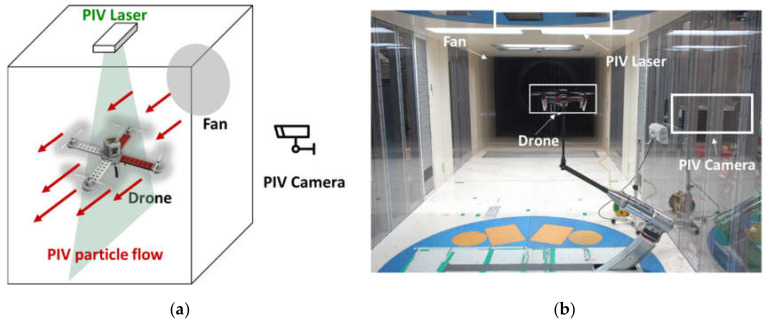
PIV experiment setup: (**a**) illustration of the observation of the aerodynamics near the UAV, and (**b**) photograph of the actual experiment setting.

Figure 4a,b shows a schematic and actual image of the PIV experiment setup. The dimension of wind tunnel used in this experiment is 3.0 m (Width) × 2.25 m (Height) × 8.75m (Length). The drone was fixed at the center of the wind tunnel for PIV analysis and PIV tracer particles were injected into the wind tunnel. The four rotors of the drone were then operated to generate a flow field and the PIV measurements were performed using the equipment settings. The wind speed (air speed) around the drone was zero while the drone rotor rotational speed was set to 5400 rpm, based on observations from the filming of the drone during an actual flight using a high-speed Phantom V6 camera. These measurements therefore allowed us to acquire the velocity and vorticity distributions around the quadrotor drone under hover flight conditions.

The PIV experiment was conducted with the assumption that the size of the sensor was sufficiently small to negligibly affect the overall air flow. The location of a sensor on a quadrotor drone is not a trivial issue and its effect requires aerodynamic analysis [43]. After sufficient filming of 300-ms movements of the tracer particles by the PIV camera, an analysis software (DynamicStudio (Dantec, Inc., Skovlunde, Denmark) which can measure and post-process analysis of the PIV data) was used to plot the average velocity vectors, which were used to roughly estimate the wind strength during the flight of a drone. In this investigation, we used average correlation method to calculate average flow field in 600 PIV images for each operation. The velocity field distribution also enabled calculation of the pressure distribution, and vorticity distribution. Post-processing of the PIV results was also conducted using ‘Tecplot 360’ software made by Tecplot, Inc, Bellevue, WA, USA.

Vorticity is related to the local angular velocity of the flow, and hence is used as an indication of the presence of vortices. The vorticity distribution was calculated from the velocity field using Tecplot 360. The ultimate objective is to figure out stable location for the chemicapacitance sensor during realistic drone operation. For the objective, we measured the velocity and vortex effects through PIV analysis and compared the sensor performances of different location on our drone system. 

## 3. Results

### 3.1. Aerodynamic Fields around the Drone

#### 3.1.1. Velocity Field around the Drone

**Figure 5:**
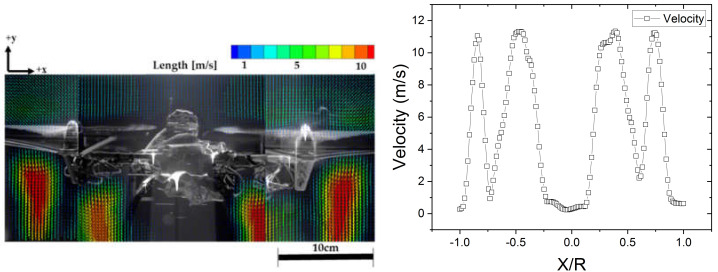
Vertical distribution of the velocity field around the drone: (**Left**) visualization and (**Right**) magnitude with respect to rotor spacing.

Figure 5 shows the vertical velocity distribution around the quadrotor drone with respect to the rotor spacing for the rotor at *X* = [−1.0*R*, 1.0*R*], *Y* = −0.5*R* (the origin is the center of the drone) and the *Z* position of the PIV plane is *Z* = 1.3*R* (across the center of the rotors). As can be observed from the figure, the flow is concentrated under the lower surfaces of the rotors. This is evident from the positions of the flow field peaks; the velocity within the wake boundary and the radial outward expansion of the rotor-induced flow produce nine peaks, namely four extreme maxima and five extreme minima. The extreme maxima occur at *X* = −0.85*R*, −0.48*R*, 0.49*R*, and 0.85*R*, while the extreme minima occur at *X* = −1.0*R*, −0.75*R*, 0.0*R,* 0.70*R*, and 1.0*R*. Furthermore, Figure 5 indicates that the air flow generated by the drone’s flight is strong below the rotors. The air velocities above the rotors are almost zero (below 1–4 m/s), while strong flows with velocities above 10 m/s develop under the rotors. The areas below the rotors are characterized by a steep velocity gradient. Particularly, in the areas around the rotors where the velocity gradient rapidly changes within a narrow region (near −0.75*R* and 0.75*R* in Figure 5), the velocity distribution consists of two adjacent extreme maxima enclosing an extreme minimum. These observations are consistent with the results of a previous analysis of the interference wake of twin rotors [53]. The most typical feature in the regions of the rotors was a high vorticity, and information could be obtained about the wake development in specific regions through analysis of the vorticity [54], including the vorticity distribution.

#### 3.1.2. Vorticity Distribution

**Figure 6:**
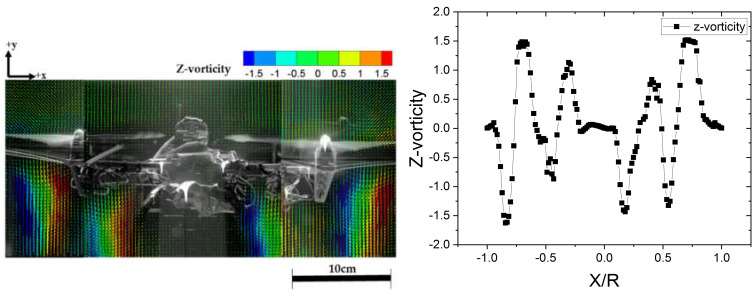
Vertical vorticity distribution around the drone (**Left**) visualization and (**Right**) magnitude graph.

The vorticity field under the interaction between the drone rotors is shown in Figure 6, as determined by PIV. The tangential velocity of the propeller inflow under the effect of the vortex can be examined from Figure 5 and Figure 6. As can be observed from the figures, the distributions of the vorticity and velocity are similar. The vortex generated from the rotor tip was analyzed based on the PIV measurements in the lower panel of Figure 6. The separation distance between the rotor tips was 68% of the rotor diameter (0.68*D*).

The average location of the rotor wakes is 0.5*R* from the drone axis downstream of the propeller. The mean flow moves radially inward as they pass through the propeller, while they circumferentially move in the same direction as the rotation of the slipstream, with a displacement of about 0.68*R* in the measurement plane. The radially inward motion of the rotor wakes may be primarily ascribed to the contraction of the stream tube, while the circumferential motion is mainly due to the tangential velocity of the slipstream, i.e., the swirl.

In the vorticity distribution shown in Figure 6, the positive values (red-colored vectors) correspond to the counter-clockwise vorticity, while the negative values (blue-colored vectors) represent the clockwise vorticity. The vorticity distribution is point-symmetric with respect to 0.0*R*. The maximum and minimum vorticity magnitudes are 1.5 and 0, respectively. The central axis of the drone, where velocity magnitude and vorticity magnitude are low, as shown in Figure 5 and Figure 6, therefore represent a relatively calm region of air flow.

If various chemical agents such as DMMP are clustered near the drone, they would either have enforced outside the streamtube by the strong outflow that develops downstream, as indicated in Figure 5, or transported via the inflow of the streamtube. The transported materials would be dissipated around the drone owing to the turbulence formed by the rotors. The effect of the vortices on the performance of the propellers was analyze through the propeller local inflow, i.e., the tangential and axial velocities, as well as the out-of-plane force and moment of the propeller.

**Figure 7:**
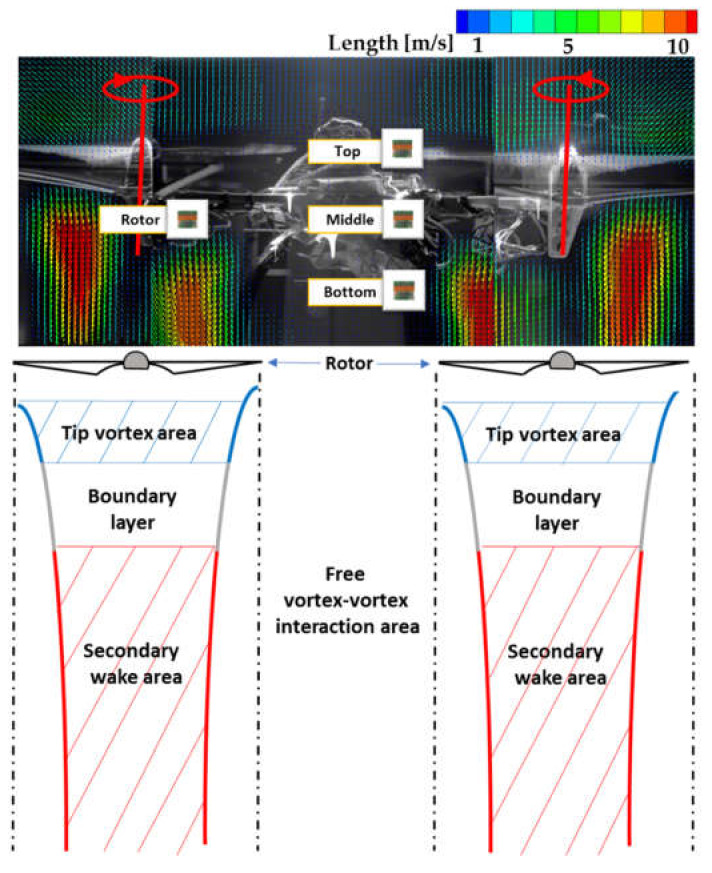
Vortex phenomena caused by the differing flow rates in two adjacent sections under the rotor blades. Although the tip vortex area and boundary layer seem to be fairly thick from the illustration, they are actually very thin, and the secondary wakes constitute most of the flow profiles. Also, based on the present PIV measurements, we selected four possible sensor locations (Rotor, Top, Middle and Bottom) on a drone.

Figure 7 shows the wake layout of a two-bladed rotor, with the wakes of the two rotors distinguished by blue and red, respectively. The blue-lined areas represent the tip vortex areas, which contain the tip vortex formed by the rotor tip and represent the primary wakes. A primary wake is a vortex that occurs immediately after a rotor is rotated. The tip vortex area consists of a small layer formed just below the lower surface of the rotor [55]. The gray-lined areas represent the boundary layer of the stream tube under the rotors, separating the tip vortex area and secondary vortex area. The flow here is not turbulent but laminar with a low flow rate and pressure, and is the main generator of the drone lift. Studies have shown that the shear stress between the air flow and the propeller continue to act in this area, resulting in momentum and energy exchanges [56,57,58]. The presence of this boundary also generates noise around the propeller and fuselage, where the noise is highest owing to the concentration of the lift [59]. The red-lined areas represent the secondary vortex area, which is where the secondary wakes from the tip vortices mix to form new wakes. The primary aerodynamic flow of the drone is dominated by two vortices on the upper surface of the blade in the vicinity. The counter-rotating vortices are caused by the pressure on the lower surface of the wing being higher than that on the upper surface.

Thus, the lower part of the rotor is where the air flow momentum changes, pressure changes, and lift distribution causes instability. The areas where these vortex phenomena do not occur are relatively stable. Due to the relatively large gap between the rotors (>0.5*D*), the vortex phenomena do not occur in the gap area, which is referred to as a vortex–vortex interaction-free area [34]. For the drone used in this study, the middle area was also the vortex–vortex interaction-free area because the distance between the propellers was 0.68*D*.

The pressure and turbulence distributions above and below the rotors are different [38]. Therefore, it is meaningful to compare the sensor performances at different points in a wake-free area by demarcating the middle area into top, center, and bottom parts. Hence, we selected a total of four comparative points, consisting of the foregoing three with a fourth point on the rotor, and conducted the following sensor performance tests.

### 3.2. Sensor Performance Test

Previous studies have shown that the air flows produced by the rotors can affect sensor measurements. Based on the present PIV measurements and quadrotor aerodynamics, there are four possible sensor locations on a drone: the central part of the top of the drone body (Top), the central part of the side of the drone body (Middle), the central part of the bottom of the drone body (Bottom), and the bottom surface of the rotor (Rotor).

**Figure 8:**
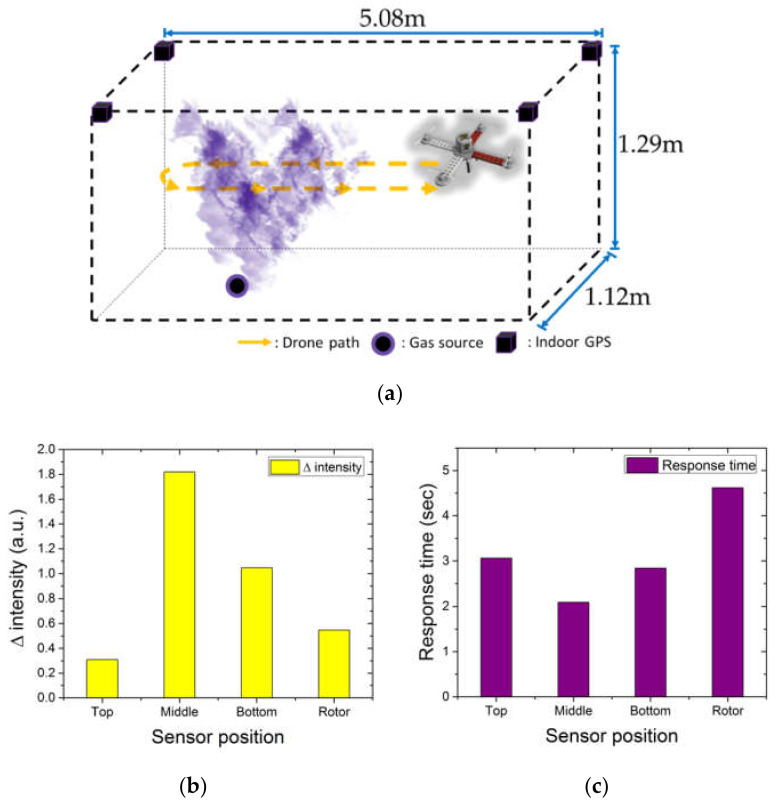
(**a**) Schematic of comparative sensor performance test, and (**b**) average capacitances and (**c**) sensor response times to DMMP at four different sensor positions on the drone.

We combined a CNT sensor with the quadrotor drone to develop a DMMP detection system and tested it indoors (Figure 8a) to optimize the sensor position and orientation for maximum detection performance under the effect of the rotor-induced air flow. Based on a previous PIV study, we confirmed that the present drone had a stable air flow region beside the main frame during operation. Hence, the specific purpose of the indoor test was to identify the optimal sensor position and orientation beside the main frame. The data and drone trajectories recorded during the tests were used to determine the sensor response times at the different considered installation points. Firstly, we conducted basic DMMP detection tests by moving the drone forward and backward. The walk-in hood system used for the tests had an interior space that measured 5.08 m × 1.12 m and a height of 1.29 m. The DMMP gas was uniformly exhausted by the ventilation fan of the hood system and the drone was operated with a rotor speed of 5400 rpm and flight speed of 0.4 m/s in the DMMP zone. The drone with the fitted CNT sensor began its fight in the refresh zone, entered the DMMP zone, and then returned to the refresh zone, as illustrated in Figure 8a.

Repeated forward and backward tests confirmed that the gas generation was uniform, and the sensor fully recovered its original capacitance when it returned to the refresh zone. The tests were repeated using different sensor locations. The direction that the sensor faced when the drone entered the gas zone was always maintained perpendicular to the floor and each measurement was conducted three times. 

The results showed identical trends of the sensor sensitivity to response intensity and the response time of the measurements for the different sensor positions. Through direct experimentation, we confirmed the variability of the velocity field around the drone which were observed through PIV. The capacitance change (Δ intensity) and response time were determined as the average of three measurements for each position. The measurement results revealed that the capacitance change in Figure 8b, which represents the maximum sensitivity on exposure to the same gas concentration for the same time, was highest for the Middle sensor position, followed by the Bottom, Rotor, and Top positions, respectively. The highest capacitance change indicated that the highest density of the chemical agent was transported during gas exposure. In addition, a higher capacitance change implied a region with a lower degree of disturbance of the air by the drone rotor and of scattering by turbulence. Within the vortex-free area, the middle and bottom parts had higher performance values compared with the rotor, which was the region with the highest flow rates, while the values at the top were lower. The smaller transport in the top region can be attributed to the air flow generated upstream of the rotor in the downstream direction. This is evident from the significant difference between the flow velocities above and below the rotor, as indicated in Figure 5. This phenomenon is also considered to be indicative of the absence of upward air flow, because almost no vortex is generated in the upper region. This can be observed from a comparison of the vorticities in the upper and lower regions of the rotor in Figure 6.

In Figure 8b, the response time is calculated by measuring the time from the initial detection of DMMP upon entry into the gas zone to extreme minimum capacitance of DMMP signal. The shortest response time was observed for the Middle senor position, followed by Bottom, Top, and Rotor, respectively. A shorter response time also indicates a faster transport of the chemical agent under given conditions. The sensor performances in the Middle and Bottom positions are higher because they fall within the vortex-free area, which facilitates transport of the chemical agent to the sensor without dissipation by turbulence. However, at the Top and Rotor positions, the higher capacitance change was observed at the Rotor, but the shorter response time observed at the Top (i.e., vortex-free area) owing to direct material transport without dissipation. The capacitance change is a measure of the amount of material transported within a given time and the results for the Top position in comparison with Rotor can therefore be attributed to the inability to overcome the strongly developed downstream flow.

In addition to the response intensity and response time, another important indicator is the response time error for each sensor location in described in Table 2. At the Middle position, which had the highest values of the previous two measures, the error was lowest at 0.1045, while it was highest at the Rotor (0.2310). This indicates that the sensor response time is unstable, with the Rotor position producing the poorest uniformity of chemical detection, and the Middle position the best.

## 4. Discussion

In various researches, many trials were conducted to observe air flow effect and velocity field around the drone system [23,60]. Several reports have reported the affect that chemical sensor performance would have on the air flow effect and velocity field due to air stream change [14,44,61]. Mostly, chip-sized chemical sensors such as chemi-resistor and chemi-capacitance sensors are very sensitive to the air flow effect, due to their basic principle of chemical detection being based on the chemical adsorption of detection material on chemical sensing channel [30,62]. For this reason, installment of sensors at a suitable location whose air flow is relatively stable is very important to reduce the false alarm of chemical sensor. Based on the PIV analysis, we chose a stable location for the chemi-capacitance type sensor at the middle location in our customized drone. In addition, we focused on observation the relationship between air flow from the rotors and sensor performance.

We successfully customized the chemical detectable drone system by embedding pixhawk software and installing chemical sensor to monitoring gas leakage point. We tried to conduct comparison tests to find suitable location of chemicapacitance sensor on drone system to reduce the external flow effect from rotors. Through the PIV analysis, four different sensor locations were selected, and we confirmed the stable sensing performance from the Middle location. 

In this study, four different locations were selected based on PIV analyses of the velocity and vorticity fields, and their distinct DMMP detection performance were successfully measured in regulated environment through large fume hood facility. We confirmed that feasibility of drone as chemical reconnaissance platform and relationship between locations and air flow effect based on PIV analysis. However, further study to investigate the sensor performance under more realistic outdoor conditions still needs to be undertaken.

## 5. Conclusions

We investigated the feasibility of using a quadrotor drone in a system for detecting chemical agents in the environment, such as in chemical, biological, radiological, and nuclear reconnaissance in the military and other industries. Below is a summary of the study and the findings. 

The air flow velocity vector field was visualized by PIV under hover flight conditions of a 69-cm-wide quadrotor drone fitted with a CNT sensor.

The obtained vector field was used to determine the velocity gradient distribution, which verified regions of higher and lower air velocity.

The vorticity distribution was also obtained by calculation from the flow velocity vector field and used together with the velocity distribution to analyze the location of the rotor wakes. The analysis indicated that the axis of the drone was relatively free of rotor effects.

Based on comparative analysis on the instabilities at different points on the quadrotor drone using the velocity and vorticity distributions, sensor performance tests were conducted with the CNT sensor attached at four locations, namely on the rotor and on the top, at the middle, and at the bottom of the drone body.

The four sensor locations produced different response intensities and response times. Location on the rotor produced the poorest results for both metrics owing to the high instability in the region, while the sensor sensitivity was highest for the middle location where the instability was the lowest.

Our study can be summarized as follows:

- Visualization of the effect of the flow field around the drone
- Determination of the effect of the customized quadrotor drone structure on the flow field, and hence the magnitude of the effect of the fluid field on the chemical detection
- Adaptation of the direct drone aerodynamics feedback to a realistic experiment
- Demonstration of the feasibility of using a quadrotor drone for chemical detection.

## Figures and Tables

**Table 1:** Specifications of customized drone.

Parameter	Quantity
**Weight**	1.8 kg
**Size**	Diagonal: 45 cm, prop diameter: 12 cm, height: 25 cm
**Payloads**	LiDAR, CNT sensors, indoor GPS system, Telemetry, Flight controller
**Communication band**	915 MHz
**RC frequency**	2.4 GHz
**Propulsion**	4 brushless electric motors
**Speed**	0 to 18m/s
**Flight controller**	PX4 (model: Pixhawk 2)
**Control Interface**	GCS: Laptop, Software: QgroundControl (Dronecode Project, Inc.)

**Table 2:** Response time measurement errors.

	Top	Middle	Bottom	Rotor
Response time error	0.1531	0.1045	0.1420	0.2310
